# The Prevalence of Modifiable Parental Behaviors Associated with Inadvertent Pediatric Medication Ingestions

**DOI:** 10.5811/westjem.2018.12.40952

**Published:** 2019-02-11

**Authors:** Matthew Salzman, Lia Cruz, Sandra Nairn, Samuel Bechmann, Rupa Karmakar, Brigitte M. Baumann

**Affiliations:** * Cooper Medical School of Rowan University, Department of Emergency Medicine, Camden, New Jersey; † Cooper Medical School of Rowan University, Department of Pediatrics, Camden, New Jersey

## Abstract

**Introduction:**

Our aim was to examine potential risk factors and modifiable behaviors that could lead to pediatric poisonings. Our secondary objectives were to explore socioeconomic factors associated with caregiver (parent/guardian) safe medication storage and knowledge of poison control contact information.

**Methods:**

We conducted a prospective, cross-sectional survey of caregivers of patients 2–10 years old presenting to an inner city pediatric emergency department. Caregiver and patient demographic data, prescription and nonprescription medication type, storage and when and where taken, were recorded. We used multivariable regression to explore factors associated with secure prescription medication storage and knowledge of poison control center contact information.

**Results:**

Of 1457 caregivers, 29% took daily prescription and 17% took daily non-prescription medications. Only 25% of caregivers stored their prescription medications in a secure place, and <3% stored medications in a locked drawer or safe. Of demographic and socioeconomic factors, only income ≥$80,000 was associated with storage of prescription medication in a secure place (odds ratio [OR], 2.47; 95% confidence interval [CI], 1.27–4.81). When asked how they would access poison control in case of an ingestion, the majority, 86%, had an appropriate plan. In multivariable regression, the only factor associated with knowledge of poison control center contact information was college education in the caregiver (OR 1.6; 95% CI, 1.10–2.32).

**Conclusion:**

A minority of caregivers store medications in a safe place and even fewer keep prescription medications under lock and key. The majority, however, were aware of how to contact a poison control center in case of ingestion.

## INTRODUCTION

In 2014, nearly 2.2 million human exposures were reported to United States poison control centers.^1^ Approximately 61%, or 1.3 million, were in the pediatric population (age ≤ 19 years), 88 of which resulted in fatality. Children younger than six years of age accounted for approximately half (47.7%) of all these exposures, with 16 reported fatalities.^1^ Nearly 70% of ingestions in children aged 12 years and younger were the result of inadvertently taking or being given someone else’s medication.^1^ Additionally, as a result of unintentional medication ingestions, approximately 70,000 children, with the peak incidence in two-year-olds, were evaluated in emergency departments (ED) annually, of whom approximately 12% required hospitalization.^2^–^6^

Several factors have been identified as contributing to inadvertent ingestions in the pediatric population. Supervision of young children by grandparents, use of containers for pills other than original prescription bottles, placement of medications in locations easily accessible to children (i.e., low storage areas, cabinets and refrigerators) and low socioeconomic status and education levels of the primary care giver(s) have all been implicated.^7^,^8^ Most of the existing studies report data using cohorts of children who had documented accidental ingestions– either via poison control center reporting or from EDs where the children presented. Few, if any, studies have been conducted examining the prevalence of established, inadvertent-ingestion risk factors in a general pediatric ED population, particularly one that presents to an inner city ED. 5,8,9

The objective of this study was to examine potential risk factors and modifiable behaviors of caregivers that may lead to inadvertent pediatric poisoning. We focused on caregivers of children aged 2–10 years of age, deemed to be at highest risk of unintentional ingestions.^2^,^9^,^10^ Additionally, we sought to determine parental awareness of poison control centers, and whether caregivers had a meaningful plan to contact a poison control center in the event of an inadvertent pediatric ingestion.

## METHODS

### Study Design

This cross-sectional study was conducted over 13 months, from November 2013 to November 2014 in the pediatric ED of a large, inner-city university hospital. Written, informed consent was obtained from adult participants, and children aged 6–10 years provided verbal assent. Our institutional review board approved this study.

### Setting and Selection of Participants

We conducted the study in the pediatric ED of a tertiary referral, university hospital ED, with an annual census of 78,000 total visits and 18,000 pediatric visits during the study period. The institution serves a catchment area of over two million people and is located in an inner city with one of the nation’s highest poverty rates.^11^ Patients were eligible for inclusion if they were between the ages of 2–10 and if the caregiver was primarily English speaking. We excluded children if they were critically ill or medically unstable. Caregivers were excluded if they were unable or unwilling to provide informed consent.

### Methods and Measurements

We created a structured, data collection form that asked caregivers for demographic information about the child they accompanied as well as about the caregivers themselves. Additional data collected included annual income and highest level of education of the caregiver, the use of both prescription and nonprescription medications by the caregiver accompanying the child, where and when medications were taken, and the storage of these medications. Finally, participants were queried about their prior use or contact of a poison control center and, if they needed to access this service, how they would go about doing so.

**Population Health Research Capsule**

What do we already know about this issue?

*Medication ingestions in children 12 years or younger are prevalent in the United States and are frequently inadvertent or accidental administration of someone else’s medication*.

What was the research question?

*To determine potential risk factors and modifiable behaviors of caregivers that could lead to inadvertent pediatric poisoning*.

What was the major finding of the study?

*Parents frequently store and consume medications that are easy for children to access*.

How does this improve population health?

*The results of this study suggest that further outreach and education is necessary to improve medication storage to decrease opportunities for inadvertent pediatric ingestion*.

Trained research assistants (RA) screened and enrolled eligible patients seven days a week, from 8:30 am to 11 pm. These time periods coincided with peak pediatric ED volumes. Participants were given the option of having the survey read to them or completing it on their own. This allowed us to capture patients who had difficulty reading due to vision or literacy problems. The RAs checked all surveys for completion, and any skipped items were reviewed with the participant to ensure as complete a dataset as possible. A brief medical record review was also undertaken to determine the reason for the child’s presentation to the ED (accidental ingestion [ingestion of a medication or another item] vs another chief complaint).

### Outcome Measures

We report percentage of caregivers who took prescription and nonprescription medications, where these medications were taken and stored, and when they were taken. We also report caregiver knowledge of poison control center contact information. We used multivariable analysis to explore factors associated with placement of prescription medications in a secure place. A secure place was defined as the following: above the countertop in the kitchen (not including above the refrigerator, as the size of appliances varies); in a medicine cabinet (above countertop height); or in a locked drawer or safe. Multivariable regression also explored factors associated with a reliable means of obtaining contact information for the poison control center. Obtaining this information from a bottle (either the medication bottle or a household product bottle) was not considered a viable method of contact. Likewise, contacting friends, neighbors or a family member were not considered appropriate resources. Contacting a healthcare provider, calling 911 or 411, referring to a poison control center magnet, a posted number, the internet, or the poison control center number added to phone memory were considered appropriate resources.

### Data Analysis

We analyzed data using Statistical Package for the Social Sciences, version 20.0 (IBM Corporation, Armonk, New York). Continuous data are presented as means with standard deviations (SD) if normally distributed and medians with interquartile ranges (IQR) for nonparametric data. Categorical data are presented as frequency counts and percentages with 95% confidence intervals (CI). Univariate analysis of categorical variables was performed using chi-squared or Fisher’s exact test, as appropriate. We conducted multivariable analysis to determine the following: 1) factors associated with placement of prescription medications in a secure place; and 2) factors associated with a reliable means of obtaining contact information for the poison control center contact information. Of note, a “secure” place was considered a cabinet above the counter or a locked drawer or safe. If a caregiver responded with multiple sites, and one site was not considered “secure,” then this caregiver was categorized as placing prescription medications in a non-secure site. Variables explored for inclusion in the regression models were selected a priori and included more than one child in the household, caregiver age, annual household income, caregiver level of education, and number of prescription medications being taken by the caregiver. Odds ratios with 95% CIs are presented. All analyses were two-tailed and *p* values<0.05 were considered statistically significant.

## RESULTS

Of 32,734 screened patients, 2007 were eligible based on age and 1495 participants were enrolled. Of the 512 who were not enrolled, 253 declined to participate or had no caregiver to provide informed consent, 136 were unable to provide informed consent due to limited English, and 123 were too ill. Characteristics of both children and their caregivers are provided in Table 1. For the majority of children, the caregiver accompanying them was the child’s mother. The mean age of the child’s caregiver was 31.1 years. In the course of the analysis, we determined that in a small percentage of cases children were brought in by a parent who did not live with the child– typically the father. For the remainder of the analysis, we only included adults who both accompanied the child and also reported living with the child, yielding a sample of 1457 children and their parents/guardians.

Nearly one-third (n=419) of the adults included in the analysis reported taking a prescription medication on a daily basis, and 251 reported taking an over-the-counter (OTC) medication on a daily basis. Of those taking a prescription medication, 70 (16.7%) reported that they did not know all the names of their medications. And when asked to list all prescription medications, 95 (22.7%) listed at least one medication as “unknown.” The median number of prescription medications taken was 1.5 (IQR:2), with a range of 1–20. For those caregivers who were taking nonprescription medications, the median of nonprescription medications was one (IQR:1) with a range of 0–6. Prescription and nonprescription medications are presented in Table 2.

Storage of prescription and nonprescription medications is presented in Table 3. The places most commonly used for storage (open-ended question on our questionnaire) are noted here, with some caregivers noting more than one location. In addition to the sites noted in Table 3, other unique sites included “under the bed,” “in the attic,” and “on the couch” for prescription medications, and “all over the house,” “no set place,” “under the bed,” “lunch bag,” and “buy it and carry it on me” for nonprescription medications. Use of the original container in which the medication was dispensed was common for both prescription and nonprescription medications, and other containers used for storage are noted in Table 3. The majority of caregivers reported taking their prescription medications (n=316, 75.4%) and their nonprescription medications (n=144, 57.3%) around the same time every day. For those taking prescription medications, 149 (35.6%), 58 (13.8%), and 88 (21.0%) took their medications at breakfast, lunch and dinner, respectively. For those using nonprescription medications, 98 (39.0%), 45 (17.5%) and 56 (22.3%) took their medications at breakfast, lunch and dinner, respectively. Approximately half of both groups, 237 (56.6%) (prescription) and 124 (49.4%) (nonprescription) did not take their medications with a meal. Locations of where medications were administered are noted in Table 3, with some respondents noting more than one location.

Caregivers of children were also questioned about prior concerns of accidental ingestions and how these were managed. These responses are noted in Table 4. When asked how they would contact the poison control center in case of an accidental ingestion, the majority, 1,248 participants (85.7%), had an appropriate plan– they had the number posted, would call 911 or 411 or a healthcare provider, planned to use the internet to find the number, or had the poison control center number entered into their cellphone/telephone. The remainder were not as organized: 116 (8%) planned to obtain the number from a medication bottle or from a household product bottle, and 93 (6.4%) participants either had no idea how to obtain the number or planned to ask a neighbor, family member or a friend.

Only 104 of 419 (24.8%) caregivers reported storing their prescription medications in a secure place. After multivariable regression, factors that remained associated with placing prescription medications in a secure place were age 30 years or older, income $80,000 or higher, and some college education or higher. Results of both univariate analyses (unadjusted) and multivariable analysis (adjusted for all five variables) are presented in Table 5. After multivariable regression, factors that remained associated with knowledge of poison control center contact information or a reliable method of obtaining it included age 30 years or older, income $80,000 or higher, and some college education or higher (Table 6).

We also examined our data to determine at what caregiver age we would reach a percentage level at which at least 35% of adults were using a prescription medication on a daily basis. At 30 years and older, 36.1% were using a prescription medication on a daily basis compared to only 20.9% of caregivers 29 years and younger (p<0.0001). For nonprescription medication use on a daily basis, the difference was not as pronounced, with 19.9% of caregivers 30 years and older vs 14.5% of caregivers aged 29 years and younger using a nonprescription medication on a daily basis (p=0.007). Finally, of all the children enrolled, 11 presented with a chief complaint of an accidental ingestion. Five children were brought in due to concerns about a potential or real ingestion of a medication, three children swallowed a toy, two swallowed a coin, and one swallowed a seed.

## DISCUSSION

Our study sought to determine the prevalence of risky and modifiable behaviors of caregivers that could lead to inadvertent pediatric poisoning. Appropriate medication storage plays an important role in minimizing inadvertent ingestion, yet 11.9% of respondents reported keeping their prescription medications on a nightstand or bedroom dresser and 21% reported storing prescription medications in a pocketbook. Both of these storage methods could easily be accessed by a child and are unsafe. In contrast, only 2.9% of respondents reported storing prescription medications in a locked drawer or safe, likely the safest storage methods for prescription medications (Table 3). Our data replicate findings by McFee et al. in their study of children’s inadvertent exposure to grandparents’ medications, where medications were placed on tables or countertops (46%), low shelves (29%), and pocketbooks (17%).^5^ While it was reassuring that the majority of our participants kept their medications in the original (presumably child-resistant) containers, this unfortunately does not appear to be a sufficient deterrent. Several studies have shown that child-resistant containers are almost as frequently involved in inadvertent pediatric poisoning as “easy open” containers.^3^,^5^,^8^ It appears the location of the storage– easily accessible locations vs locked or elevated locations– may play a greater role in inadvertent pediatric ingestions.^5^, ^8^

A concerning finding was the location where caregivers ingested their medications. Over half were consumed in the kitchen or dining room, locations which could easily lead to inadvertent ingestion of a lost or misplaced pill by a child, simply due to the fact that there is a greater likelihood of children being present, allowing them to grab and ingest the caregiver’s pills. This also proposes a new association to the child who is now visualizing a caregiver ingesting a pill in a location with which they associate mealtime and being instructed to eat.

An additional concerning finding was that over 10% of caregivers reported taking their medications “on the go” or in the car. This is problematic as distractions can easily interfere with proper oversight and storage of medications.

We found that potential medications that the children in our study could have been exposed to closely mirrored those found in other investigations of actual pediatric exposures.^2^,^4^,^5^ Medications most commonly reported to be used by caregivers included analgesics, cardiovascular drugs, anticonvulsants, and psychotropic medications. All of these pharmaceuticals have the potential for significant morbidity and mortality if ingested by a small child. This presents an additional educational opportunity to provide to caregivers of small children. In addition to enforcing safe medication storage and avoidance of associating medication ingestion with mealtimes and locations of food consumption, introduction to the concept of high-risk medications and the fact that OTC medications can have devastating adverse effects is also important.

Other novel findings in our investigation included caregivers’ reports of prior experiences with near or actual ingestions and their response to these. While nearly two-thirds appropriately managed their children, seeking care at the ED or from their pediatricians, over 15% did not seek any formal medical care or advice. Equally concerning was that 212 (12.6%) of caregivers reported having called a poison control center for a possible ingestion at some time. This suggests that, at least in our inner city population, there is a real risk of potential ingestion and, as others have recommended, additional parental education for both prevention and access to medical care/guidance is needed in the event of an ingestion.^2^,^4^,^5^

Lastly, we attempted to determine demographic characteristics associated with placing prescription medication in a safe place as well as with knowledge of poison control center contact information. Multivariable analysis determined that only annual income greater than $80,000 was associated with placing prescription medications in a safe place. In our second regression, only a higher level of education was significantly associated with knowledge of poison control center contact information. We expected older age and having more than one child (more mature, more life experience) and, especially, higher education level to be strongly associated with both factors. We are unsure why this relationship is lacking. In their sample of parents of children aged 1–6 years admitted to the University of Puerto Rico Carolina Hospital pediatric ward, Gutierrez, et al. also demonstrated that educational level was not related to a lack of knowledge in how to contact a poison control center but did not offer any explanation for why this occurred.^9^

## LIMITATIONS

This was a single-site investigation in an inner city ED, so our results may not be generalizable to other patient populations. The patient sample surveyed in this study was a convenience sample, with recruiting only when academic associates were present. However, as previously noted, this time encompassed the peak hours for pediatric ED visits. Given the nature of this questionnaire there may have been issues with social desirability bias, as we relied on self-report of where and how medications were stored. We also relied on self-report in caregivers remembering all medications they were taking and, furthermore, their impression of what is considered a medication. Likewise, the validity of self-reported knowledge of poison control center contact information may also have been biased. Caregivers may not have contact information posted or may not have input the number into their cellphones but may have responded as such.

Further, because this is a survey study based on previous behaviors, responder recall bias may be a substantial limitation. Finally, due to language constraints, we were not able to enroll many non-native English speakers, a population that may have its own unique characteristics with respect to risk factors for inadvertent pediatric ingestions. In our department, our non-English speaking patients are primarily Hispanic, and many are migrants or immigrants. Given the language barriers, socioeconomic constraints and unique cultural practices with home remedies (some of which incorporate lead), we suspect that this missed population is particularly vulnerable to accidental ingestions and at greater risk for adverse outcomes due to limited access to healthcare guidance and management.

## CONCLUSION

In our inner city ED, parents continue to store and consume medications in locations that are easy for children to access. While many have an appropriate plan for reaching the poison control center in the case of an inadvertent ingestion, a sizable minority do not. This is of particular concern, since over 15% of our caregivers had already obtained poison control center assistance in the past, suggesting that the incidence of inadvertent ingestion is fairly high. Of those who actually were concerned about a potential inadvertent ingestion by a child, nearly 18% did not seek any medical advice or intervention. These data suggest that further outreach and education is necessary to improve medication storage by caregivers and increase poison control center awareness.

## Figures and Tables

**Table 1:** Characteristics of participants (N=1,495).

	n (%)
Age of child, mean (SD)	*5.6 (2.7)
Male	783 (52.4)
Female	712 (47.6)
Race	
White	745 (49.8)
Black	700 (46.8)
Asian	22 (1.5)
Other	28 (1.9)
Hispanic	675 (45.2)
Child’s primary caregiver	
Mother	1,301 (87.0)
Father	150 (10.0)
Grandmother	27 (1.8)
Aunt	5 (0.3)
Other	12 (0.8)
Person accompanying child	
Mother	1,266 (84.7)
Father	180 (12.0)
Grandmother	30 (2.0)
Aunt	7 (0.5)
Other	12 (0.8)
Mean age of person accompanying child (SD)	*31.1 (0.8)
Child lives with	
Mother only	711 (47.6)
Father only	24 (1.6)
Both mother and father	639 (42.7)
Grandmother	25 (1.7)
Mother and grandmother	3 (0.2)
Other	93 (6.2)
Child accompanied by someone who lives with him/her	1,457 (97.5)
Annual income of adult who accompanied the child and who also lives with child	(n=1,457)
≤$20,000	651 (44.7)
$20,001–$40,000	420 (28.8)
$40,001–$60,000	131 (9.0)
$60,001–$80,000	86 (5.9)
≥ $80,001	105 (7.2)
Declined to answer	64 (4.4)
Level of education of adult who accompanied child and who also lives with the child	(n=1,457)
Did not graduate high school	188 (12.9)
High school graduate	531 (36.4)
Vocational/ tech school graduate	92 (6.3)
Some college	378 (25.9)
College graduate	255 (17.5)
Declined to answer	13 (0.9)
Mean number of children who live in the home 18 years and younger (SD)	2.4 (1.2)

*SD*, standard deviation.

* years.

**Table 2:** Medications used by participants who live with the child (N=1,457).

	n (%)
Analgesics	
Acetaminophen	58 (4.0)
NSAIDs	64 (4.4)
Opiate analgesics	31 (2.1)
Aspirin	21 (1.4)
Tramadol	15 (1.0)
Cardiac and antihypertensive medications	
Beta blocker	26 (1.8)
Diuretic	25 (1.7)
ACE inhibitor/ARB	23 (1.6)
Calcium channel blocker	13 (0.9)
Angiotensin receptor blocker	7 (0.5)
Clonidine	1 (0.1)
Warfarin	1 (0.1)
Other blood thinners	1 (0.1)
Diphenhydramine	13 (0.9)
Diabetes medications	
Metformin	15 (1.0)
Sulfonylurea	5 (0.3)
Psychiatric medications	
TCAs	1 (0.1)
Antipsychotics	8 (0.5)
SSRIs	63 (4.3)
Seizure medication (excluding barbiturates)	30 (2.1)
Other controlled substances	
Benzodiazepines	21 (1.4)
Barbiturates	10 (0.7)
Supplements	
Multivitamin	147 (10.3)
Iron tablets	19 (1.3)
Fish oil	11 (0.8)

*NSAID*, non-steroidal anti-inflammatory drug; *ACE*, angiotensin-converting enzyme; *ARB*, angiotensin receptor blocker; *TCA*, tricyclic antidepressant; *SSRI*, selective serotonin reuptake inhibitor.

**Table 3:** Storage of medications and location where medications are taken by participants who live with the child.

	Prescription medications; n=419	Non-prescription medications; n=251
		
	n (%)	n (%)
Storage location		
Kitchen cabinet above counter	99 (23.6)	97 (38.6)
Medicine cabinet in bathroom	97 (23.2)	71 (28.3)
Pocketbook	88 (21.0)	20 (8.0)
On nightstand or dresser in bedroom	50 (11.9)	9 (3.6)
In bedroom in a drawer	42 (10.0)	20 (8.0)
In a closet	20 (4.8)	15 (6.0)
Locked drawer or safe	12 (2.9)	2 (0.8)
Kitchen cabinet below counter	7 (1.7)	7 (2.8)
Top of refrigerator	4 (1.0)	6 (2.4)
On countertop	6 (1.4)	3 (1.2)
In the refrigerator	4 (1.0)	2 (0.8)
In the car	3 (0.7)	1 (0.4)
Windowsill	1 (0.2)	2 (0.8)
Container storage		
Original bottle	411 (98.1)	238 (94.8)
Plastic dispenser or pill box	17 (4.1)	7 (2.8)
Loose	1 (0.2)	4 (2.0)
Plastic bag	2 (0.5)	1 (0.4)
Use of child-resistant cap for prescription medications	373 (89.0)	---
Location where medications are taken		
Kitchen	198 (47.3)	144 (57.4)
Bedroom	121 (28.9)	62 (24.7)
Bathroom	95 (22.7)	37 (14.7)
At work	42 (10.2)	20 (8.0)
Anywhere/on the go	30 (7.2)	9 (3.6)
Dining room	18 (4.3)	11 (4.4)
Car	19 (4.5)	5 (2.0)
Living room	15 (3.6)	5 (2.0)

**Table 4:** Prior experience with near or actual ingestions (n=1,457).

	n (%)
Has this child ever put a pill in his/her mouth (not one that the child was intentionally given)?	
No	1,378 (95.1)
Yes, a prescription pill	38 (2.6)
Yes, an over-the-counter medication	31 (2.1)
Yes, both a prescription and an over-the-counter medication	1 (0.1)
Unsure	8 (0.5)
Were you ever worried that your child may have accidentally swallowed pills or a medication (but you were not sure or did not witness it)?	
No worries that my child swallowed a pill by accident	1,375 (94.4)
Yes, a prescription pill	33 (2.3)
Yes, an over-the-counter pill	38 (2.6)
Yes, worried about both prescription pill and an over-the-counter pill	9 (0.6)
Unsure	2 (0.1)
If you were concerned, what did you do?	(n=80)
Emergency department visit	29 (36.3)
Called poison control	18 (22.5)
Observed child at home (without medical guidance)	10 (12.5)
Called pediatrician	6 (7.5)
Did not do anything	4 (5.0)
Pediatrician visit	1 (1.3)
Called poison control for any possible ingestion for anyone?	212 (14.6)

**Table 5:** Factors associated with placing prescription medications in a secure place (locked storage or above countertop). Multivariable analysis (n=419).

	Unadjusted odds ratio (95% CI)	P value	Adjusted odds ratio (95% CI)	P value
More than one child in family	1.74 (0.99–3.07)	0.55	-	-
Age 30 years and older	1.89 (1.15–3.12)	0.013	1.48 (0.87–2.51)	0.145
Income $80,000 or higher	3.28 (1.77–6.09)	<0.0001	2.47 (1.27–4.81)	0.008
Some college education or higher	1.89 (1.18–2.90)	0.007	1.40 (0.87–2.28)	0.170
Use of more than four prescription medications	1.79 (0.73–4.42)	0.21	-	-

*CI*, confidence interval.

Variables included in the multivariable regression model were selected if p<0.05 on unadjusted univariate analysis.

**Table 6:** Factors associated with knowledge of Poison Control Center contact information or a reliable method of obtaining it (n=1,457).

	Unadjusted odds ratio (95% CI)	P value	Adjusted odds ratio (95% CI)	P value
More than one child in family	0.97 (0.65–1.44)	0.88	-	-
Age 30 years and older	1.48 (1.05–2.09)	0.027	1.29 (0.90–1.83)	0.16
Income $80,000 or higher	4.05 (1.27–12.94)	0.018	2.82 (0.86–9.26)	0.88
Some college education or higher	1.83 (1.27–2.63)	0.001	1.6 (1.10–2.32)	0.01
Use of more than four prescription medications	1.45 (0.58–3.68)	0.43	-	-

*CI*, confidence interval.

Variables included in the multivariable regression model were selected if p<0.05 on unadjusted univariate analysis.
